# Synchronization of the parkinsonian globus pallidus by gap junctions

**DOI:** 10.1186/1471-2202-15-S1-O17

**Published:** 2014-07-21

**Authors:** Bettina C Schwab, Hil GE Meijer, Richard JA van Wezel, Stephan A van Gils

**Affiliations:** 1 Applied Analysis, MIRA Institute of Biomedical Technology and Technical Medicine, University of Twente, 7500AE Enschede, The Netherlands; 2 Biomedical Signals and Systems, MIRA Institute of Biomedical Technology and Technical Medicine, University of Twente, 7500AE Enschede, The Netherlands; 3 Biophysics, Donders Institute for Brain, Cognition and Behavior, Radboud University, Nijmegen, The Netherlands

## 

The mechanisms for the emergence and transmission of synchronized oscillations in Parkinson’s disease (PD) still remain debated. In a previous publication [1], we argue that the external globus pallidus (GPe) has a crucial role in desynchronising and synchronizing the basal ganglia. While neural activity of the healthy GPe shows almost no correlations between pairs of neurons, prominent synchronization in the β frequency band arises after dopamine depletion. Intrinsic factors of the GPe, in particular its internal connections, could be take major roles in this synchronisation process.

We introduce pallidal gap junctional coupling as a possible mechanism for synchronization of the GPe after dopamine depletion. In a confocal imaging study, we show the presence of the neural gap junction protein Cx36 in the human GPe, including a possible remodeling process in PD patients. Dopamine has been shown to down-regulate the conductance of gap junctions in different regions of the brain [2,3], making dopamine depletion a possible candidate for increased influence of gap junctional coupling in PD.

To see what effect electrical coupling in the GPe could have, we incorporate gap junctions in a small conductance-based model of the basal ganglia. In both GPe and GPi, gap junctional coupling has clear effects on synchrony. Especially numerous coupling with sufficient strength in the GPe is able to synchronize the whole basal ganglia. Next, we focus on dynamics inside the GPe. Phase-response curve analysis is used to describe the susceptibility of GPe neurons to synchronize with input, depending on electrical coupling to other GPe neurons. Additionally, we simulate the effect of gap junctions on synchrony in a larger network of the GPe, including biologically realistic cell models and inhibitory synaptic coupling.

## Conclusions

We hypothesize that strong gap junctional coupling in the GPe disturbs the self-desynchronization in this nucleus and leads to long-range synchronization. Pallidal gap junctions, which are potentially modulated by dopamine, could be a powerful trigger of synchrony in Parkinson's disease. We stress that also gap junctions in other nuclei such as the striatum may play important roles.
